# Combination of HLA-DR on *Mycobacterium tuberculosis*-Specific Cells and Tuberculosis Antigen/Phytohemagglutinin Ratio for Discriminating Active Tuberculosis From Latent Tuberculosis Infection

**DOI:** 10.3389/fimmu.2021.761209

**Published:** 2021-11-11

**Authors:** Ying Luo, Ying Xue, Guoxing Tang, Qun Lin, Huijuan Song, Wei Liu, Botao Yin, Jin Huang, Wei Wei, Liyan Mao, Feng Wang, Ziyong Sun

**Affiliations:** ^1^ Department of Laboratory Medicine, Tongji Hospital, Tongji Medical College, Huazhong University of Science and Technology, Wuhan, China; ^2^ Department of Immunology, School of Basic Medicine, Tongji Medical College, Huazhong University of Science and Technology, Wuhan, China

**Keywords:** *Mycobacterium tuberculosis*-specific cells, HLA-DR, TBAg/PHA ratio, discrimination, active tuberculosis, latent tuberculosis infection

## Abstract

**Background:**

Novel approaches for tuberculosis (TB) diagnosis, especially for distinguishing active TB (ATB) from latent TB infection (LTBI), are urgently warranted. The present study aims to determine whether the combination of HLA-DR on *Mycobacterium tuberculosis* (MTB)-specific cells and TB antigen/phytohemagglutinin (TBAg/PHA) ratio could facilitate MTB infection status discrimination.

**Methods:**

Between June 2020 and June 2021, participants with ATB and LTBI were recruited from Tongji Hospital (Qiaokou cohort) and Sino-French New City Hospital (Caidian cohort), respectively. The detection of HLA-DR on MTB-specific cells upon TB antigen stimulation and T-SPOT assay were simultaneously performed on all subjects.

**Results:**

A total of 116 (54 ATB and 62 LTBI) and another 84 (43 ATB and 41 LTBI) cases were respectively enrolled from Qiaokou cohort and Caidian cohort. Both HLA-DR on IFN-γ^+^TNF-α^+^ cells and TBAg/PHA ratio showed discriminatory value in distinguishing between ATB and LTBI. Receiver operator characteristic (ROC) curve analysis showed that HLA-DR on IFN-γ^+^TNF-α^+^ cells produced an area under the ROC curve (AUC) of 0.886. Besides, TBAg/PHA ratio yield an AUC of 0.736. Furthermore, the combination of these two indicators resulted in the accurate discrimination with an AUC of 0.937. When the threshold was set as 0.36, the diagnostic model could differentiate ATB from LTBI with a sensitivity of 92.00% and a specificity of 81.82%. The performance obtained in Qiaokou cohort was further validated in Caidian cohort.

**Conclusions:**

The combination of HLA-DR on MTB-specific cells and TBAg/PHA ratio could serve as a robust tool to determine TB disease states.

## Introduction

Tuberculosis (TB), caused by *Mycobacterium tuberculosis* (MTB) infection, remains an ongoing and leading global public issue with high morbidity and mortality (1). It was reported that the disease caused an estimated 10.0 million incident cases and approximately 1.4 million deaths worldwide in 2019 (2). Most subjects infected with MTB remain relatively healthy, a state called latent TB infection (LTBI) (3). However, approximately 5-10% of these individuals will eventually develop to active TB (ATB) during their life (4). Rapid approaches that can differentiate ATB from LTBI are essential for TB management and control, as well as the implement of the end TB strategy (5). Thus, developing effective and feasible methods become a current priority in combating the disease.

Currently, diagnosing ATB is mainly based on identifying the pathogen by staining for acid-fast bacilli, mycobacterial culture or PCR such as GeneXpert MTB/RIF. However, these methods are either insensitive or time-consuming, failing to meet clinical needs (6). Meanwhile, two kinds of commercial interferon gamma release assays, including QuantiFERON-TB Gold In-Tube test (QFT-GIT) and T-SPOT.TB (T-SPOT), were widely used for identifying MTB infection (7). Nevertheless, both T-SPOT and QFT-GIT are intrinsically unable to discriminate between ATB and LTBI well (8, 9). To address these limitations, emerging techniques, including transcriptomics (10, 11), proteomics (12, 13), and metabolomics (14, 15) have recently been introduced. However, these technique-derived tests lack sufficient validation and are difficult to carry out in clinical practice due to cumbersome operating procedures and requirements for special equipment (16). Therefore, despite much effort to identify new diagnostic methods for TB, we still lack affordable and efficient tools, especially based on existing platform, targeting this issue.

Surface markers on immune cells and intracellular cytokines detected by flow cytometry had been applied in TB diagnostic field in recent years (17, 18). Among these efforts, the activation phenotype represented by HLA-DR on MTB-specific cells appeared to be particularly outstanding in identifying ATB (19). However, in a more recent study, Mpande et al. denoted that HLA-DR on MTB-specific cells could not differentiate ATB patients from LTBI individuals with recent MTB infection (20). Thus, HLA-DR on MTB-specific cells would show relatively moderate specificity in distinguishing ATB from LTBI. Hence, there is a considerable need to seek a method with high specificity to combine with HLA-DR to compensate for its loss in specificity. Wang and his colleagues previously developed an indicator-TB antigen/phytohemagglutinin (TBAg/PHA) ratio, which showed relatively high specificity and moderate sensitivity in discriminating ATB patients from LTBI individuals (21, 22). We wonder whether the combination of these two indicators could further improve the differentiation. Therefore, the present study aims to investigate the potential value of the combination of HLA-DR on MTB-specific cells and TBAg/PHA ratio in distinguishing between ATB and LTBI.

## Methods

### Subjects

The current study was conducted between June 2020 and June 2021. Participants were recruited from Tongji Hospital (Qiaokou cohort, the largest tertiary hospital in central China with 5500 beds) and Sino-French New City Hospital (Caidian cohort, a branch hospital of Tongji Hospital with 1600 beds), respectively. Participants in two cohorts were enrolled based on positive T-SPOT results. Patients with suspected symptoms of ATB and eventually confirmed by microbiological evidences were included. The definition for ATB was positive culture for MTB and/or positive GeneXpert MTB/RIF, as well as supportive symptoms and radiological evidence for ATB. LTBI individuals included in the current study were recruited from the populations who underwent health screening at hospitals. LTBI individuals were defined by positive T-SPOT results with no clinical or radiographic evidence of ATB (3, 23–25). Subjects were excluded if they had received anti-TB chemotherapy or younger than 17 years old. All enrolled subjects were HIV-negative. In order to determine the change of various indicators during anti-TB treatment, three months of anti-TB treatment was performed on ATB patients with isoniazid, rifampicin, pyrazinamide, and ethambutol. Three consecutive negative GeneXpert MTB/RIF results and relief of the patient’s symptoms were considered signs of effective treatment. The study protocol was approved by the Ethics Committee of Tongji Hospital, Tongji Medical College, Huazhong University of Science and Technology. All participants provided written informed consent.

### T-SPOT Assay

T-SPOT assay was performed using heparin-anticoagulated blood samples. The operation was conducted in accordance with manufacturer’s instruction (Oxford Immunotec, Oxford, UK). Briefly, peripheral blood mononuclear cells (PBMCs) were separated by Ficoll-Hypaque gradient centrifugation. Then, the isolated PBMCs (2.5 × 10^5^) were added to 96-well plates precoated with antibody against IFN-γ. There were four wells each participant: medium well (negative control), early secreted antigenic target 6 (ESAT-6) well (panel A), culture filtrate protein 10 (CFP-10) well (panel B), and PHA well (positive control). Cells were incubated for 16-20 h at 37°C with 5% CO_2_ and developed using anti-IFN-γ antibody conjugate with substrate to detect the presence of IFN-γ secreted cells. Spot-forming cells (SFCs) were counted with an automated enzyme-linked immunospot reader (CTL Analyzers, Cleveland, OH, USA). The criteria for T-SPOT results were recommended by the manufacturer (26). The test result was positive if ESAT-6 and/or CFP-10 spot number minus negative control spot number ≥ 6. The test result was negative if both ESAT-6 spot number minus negative control spot number and CFP-10 spot number minus negative control spot number ≤ 5. Results were considered undetermined if the spot number in PHA well were < 20 or if spot number in the medium well were > 10. We calculated the ratios of (a) ESAT-6 SFCs to PHA SFCs and (b) CFP-10 SFCs to PHA SFCs. The larger of the above two values was defined as TBAg/PHA ratio of one subject.

### Detection of HLA-DR on MTB-Specific Cells

**Figure 1:**
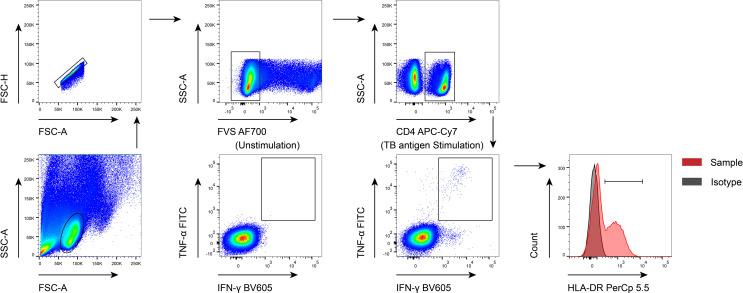
The gating strategies used in the current study. FVS, flexible viability stain; TB, tuberculosis.

PBMCs were stimulated with peptide ESAT-6 (2μg/ml) and CFP-10 (2μg/ml) for 18 hours at 37°C with 5% CO_2_. Post incubation, PBMCs were first stained with Fixable Viability Stain 700 (BD Pharmingen) to differentiate live cells from dead cells, followed by appropriate surface marker staining. Cell surface staining was performed on PBMCs using the following anti-human monoclonal antibodies: anti-CD4-APC-Cy7 (Biolegend, Clone: RPA-T4; Cat# 300518) and anti-HLA-DR-PerCp-Cy 5.5 (Biolegend, Clone: L243; Cat# 307630). For intracellular staining, the cells were fixed and permeabilized with Fixation and Permeabilization Buffer (BD Biosciences). Intracellular cytokine staining was conducted using the flowing anti-human monoclonal antibodies: anti-IFN-γ-BV605 (Biolegend, Clone: 4S.B3; Cat# 502536) and anti-TNF-α-FITC (Biolegend, Clone: MAb11; Cat# 502906). Isotype controls with irrelevant specificities were included as negative controls. After washing, the pellets were resuspended in 300μl staining buffer and analyzed with FACSCanto II flow cytometer (BD Biosciences, San Jose, CA). The flow data were analyzed using Flowjo software version 10.6.2 (TreeStar, Ashland, OR). The gating strategy was showed in **Figure 1**.

MTB-specific cells were determined by IFN-γ and TNF-α co-producing cells upon TB antigen stimulation. Responders were regarded as cases with at least ten IFN-γ^+^TNF-α^+^ recorded events, and the proportion of IFN-γ^+^TNF-α^+^ cells after TB antigen stimulation was greater than 0.03% and was at least three times the frequency of that in the unstimulating control. HLA-DR expression analysis for MTB-specific cells were only performed on responders.

### Statistical Analysis

Continuous variables were showed by median (interquartile range) or means ± standards deviation (SD). Categorical variables were expressed as number (%). Comparison between various groups was performed using Mann-Whitney *U* test for continuous variables, and Chi-square test or Fisher’s exact test for categorical variables. Wilcoxon test was used to compare various indicators of the same patient before and after anti-TB treatment. *P* values of less than 0.05 were considered significant. To establish the diagnostic model for distinguishing ATB from LTBI, variables with statistical difference were taken as candidates for further multivariable logistic regression. Subsequently, the regression equation and diagnostic model were obtained and a predictive value for each individual was calculated. Receiver operator characteristic (ROC) curve analysis was performed to define the diagnostic performance of various biomarkers. Area under the ROC curve (AUC), sensitivity, specificity, positive predictive value (PPV), negative predictive value (NPV), positive likelihood ratio (PLR), negative likelihood ratio (NLR), and accuracy, together with their 95% confidence intervals (CI), were determined. The comparison between various ROC AUCs was performed using by z test with the procedure of Delong et al. (27). All statistical analysis were conducted using GraphPad Prism software version 8.0 (GraphPad, San Diego, CA), MedCalc version 11.6 (MedCalc, Mariakerke, Belgium), and SPSS software version 25.0 (SPSS, Chicago, IL).

## Results

### Participants

A total of 116 subjects, including 54 ATB patients and 62 LTBI individuals were recruited at Qiaokou cohort. Another 84 cases, including 43 ATB patients and 41 LTBI individuals were enrolled in Caidian cohort. Demographic and clinical features of recruited participants in this study were shown in **Table 1**. The median age of included participants was around 50 years and more than half of these cases were males. No significant difference was observed between ATB and LTBI groups in distribution of age and gender among two cohorts.

**Table 1:** Demographic and clinical characteristics of recruited participants.

Variables	Qiaokou cohort (training set)		P*	Caidian cohort (validation set)		P*	*P* ^†^
	ATB (n=54)	LTBI (n=62)		ATB (n=43)	LTBI (n=41)		
Age, years	55 (35-65)	53 (32-66)	0.835	49 (29-57)	49 (37-60)	0.45	0.102
Sex, male, %	35 (64.81%)	36 (58.06%)	0.457	30 (69.77%)	28 (68.29%)	0.884	0.253
Underlying condition or illness							
Diabetes mellitus	14 (25.93%)	9 (14.52%)	0.124	9 (20.93%)	5 (12.2%)	0.283	0.57
Solid tumor	3 (5.56%)	3 (4.84%)	0.805	3 (6.98%)	2 (4.88%)	0.956	0.94
Hematological malignancy	1 (1.85%)	0 (0%)	0.466	1 (2.33%)	0 (0%)	1	1
Virus hepatitis or cirrhosis	6 (11.11%)	3 (4.84%)	0.362	7 (16.28%)	4 (9.76%)	0.376	0.214
Heart disease	5 (9.26%)	3 (4.84%)	0.569	3 (6.98%)	2 (4.88%)	0.956	0.789
End-stage renal disease	4 (7.41%)	1 (1.61%)	0.283	2 (4.65%)	1 (2.44%)	0.966	0.918
Organ transplantation	2 (3.7%)	0 (0%)	0.215	1 (2.33%)	0 (0%)	1	1
Immunosuppressive condition^‡^	6 (11.11%)	4 (6.45%)	0.575	5 (11.63%)	4 (9.76%)	0.94	0.618
Positive culture for MTB	48 (88.89%)	N/A	N/A	40 (93.02%)	N/A	N/A	N/A
Positive GeneXpert MTB/RIF	42 (77.78%)	N/A	N/A	31 (72.09%)	N/A	N/A	N/A

ATB, active tuberculosis; LTBI, latent tuberculosis infection; MTB, Mycobacterium tuberculosis; N/A, not applicable. *Comparisons were performed between ATB and LTBI groups using Mann-Whitney U test, Chi-square test, or Fisher’s exact test. ^†^Comparisons were performed between Qiaokou and Caidian cohorts using Mann-Whitney U test, Chi-square test, or Fisher’s exact test. ^‡^Patients who underwent chemotherapy or took immunosuppressants within 3 months. Data were presented as medians (25th-75th) or numbers (percentages).

### HLA-DR on MTB-Specific Cells for Distinguishing ATB From LTBI

**Figure 2:**
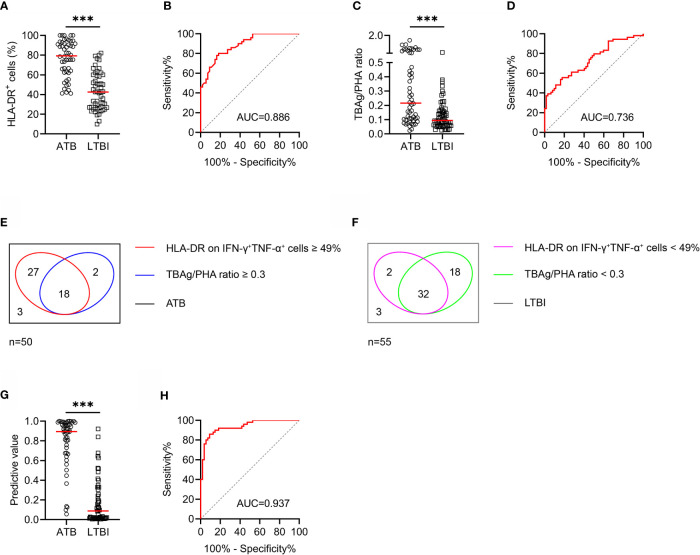
The performance of various indicators in distinguishing ATB patients from LTBI individuals in Qiaokou cohort. **(A)** Scatter dot plots showing the results of the expression of HLA-DR on IFN-γ^+^TNF-α^+^ cells in ATB patients and LTBI individuals. Horizontal lines indicate the medians. ***P < 0.001 (Mann-Whitney *U* test). **(B)** ROC curve analysis showing the performance of HLA-DR on IFN-γ^+^TNF-α^+^ cells in discriminating ATB patients from LTBI individuals. **(C)** Scatter dot plots showing the results of TBAg/PHA ratio in ATB patients and LTBI individuals. Horizontal lines indicate the medians. ***P < 0.001 (Mann-Whitney *U* test). **(D)** ROC curve analysis showing the performance of TBAg/PHA ratio in discriminating ATB patients from LTBI individuals. **(E)** Venn diagrams showing the overlap of HLA-DR on IFN-γ^+^TNF-α^+^ cells and TBAg/PHA ratio in ATB patients. **(F)** Venn diagrams showing the overlap of HLA-DR on IFN-γ^+^TNF-α^+^ cells and TBAg/PHA ratio in LTBI individuals. **(G)** Scatter plots showing the predictive value of diagnostic model in ATB patients and LTBI individuals. Horizontal lines indicate the medians. ***P < 0.001 (Mann-Whitney *U* test). **(H)** ROC curve analysis showing the performance of diagnostic model based on the combination of HLA-DR on MTB-specific cells and TBAg/PHA ratio in discriminating ATB patients from LTBI individuals. MTB, *Mycobacterium tuberculosis*; ATB, active tuberculosis; LTBI, latent tuberculosis infection; TBAg, tuberculosis antigens; PHA, phytohemagglutinin; AUC, area under the curve.

We compared HLA-DR expression on MTB-specific cells represented by IFN-γ^+^TNF-α^+^ cells upon TB antigen stimulation. It was observed that HLA-DR on IFN-γ^+^TNF-α^+^ cells was significantly higher in ATB group than that in LTBI group (**Figure 2A**). ROC curve analysis showed that HLA-DR on IFN-γ^+^TNF-α^+^ cells provided an AUC of 0.886 (95% CI, 0.826-0.946), with a sensitivity of 90.00% (95% CI, 78.64%-95.65%) and a specificity of 61.82% (95% CI, 48.61%-73.48%) to distinguish ATB from LTBI when a cut-off value of 49% was set (**Table 2** and **Figure 2B**). We stratified the subjects according to the age and found that there was no significant difference in HLA-DR expression on MTB-specific cells between individuals with young age (≤ 50 years old) and those with old age (> 50 years old) in both ATB and LTBI group (**Supplementary Figure 1A**).

**Table 2:** The performance of various indicators for discriminating ATB from LTBI in Qiaokou cohort.

Variables	Cutoff value	AUC (95% CI)	Sensitivity (95% CI)	Specificity (95% CI)	PPV (95% CI)	NPV (95% CI)	PLR (95% CI)	NLR (95% CI)	Accuracy
HLA-DR on IFN-γ^+^TNF-α^+^ cells (%)	49	0.886 (0.826-0.946)	90.00% (78.64%-95.65%)	61.82% (48.61%-73.48%)	68.18% (56.21%-78.15%)	87.18% (73.3%-94.4%)	2.36 (1.66-3.34)	0.16 (0.07-0.38)	75.24%
TBAg/PHA ratio	0.3	0.736 (0.645-0.827)	40.74% (28.68%-54.03%)	91.94% (82.47%-96.51%)	81.48% (63.30%-91.82%)	64.04% (53.69%-73.24%)	5.05 (2.05-12.42)	0.64 (0.51-0.81)	68.10%
Diagnostic model	0.36	0.937 (0.892-0.982)	92.00% (81.16%-96.85%)	81.82% (69.67%-89.81%)	82.14% (70.16%-90.00%)	91.84% (80.81%-96.78%)	5.06 (2.87-8.92)	0.1 (0.04-0.25)	86.67%

ATB, active tuberculosis; LTBI, latent tuberculosis infection; TBAg, tuberculosis-specific antigen; PHA, phytohaemagglutinin; AUC, area under the curve; PPV, positive predictive value; NPV, negative predictive value; PLR, positive likelihood ratio; NLR, negative likelihood ratio; CI, confidence interval.

### TBAg/PHA Ratio for Differentiating ATB From LTBI

The value of TBAg/PHA ratio for differentiating ATB from LTBI was also assessed. ATB patients had significantly higher TBAg/PHA ratio compared to LTBI individuals (**Figure 2C**). ROC curve analysis showed that TBAg/PHA ratio had an AUC of 0.736 (95% CI, 0.645-0.827) for discriminating patients with ATB from LTBI individuals (**Figure 2D**). When 0.3 was used as the threshold, the sensitivity and specificity of TBAg/PHA ratio for distinguishing ATB from LTBI was 40.74% (95% CI, 28.68%-54.03%) and 91.94% (95% CI, 82.47%-96.51%), respectively (**Table 2**). We stratified the subjects according to the age and found that the values of TBAg/PHA ratio in individuals with young age (≤ 50 years old) were slightly higher than those with old age (> 50 years old) in both ATB and LTBI group. However, there was no significant difference (**Supplementary Figure 1B**).

### Establishing Diagnostic Model Based on Combining MTB-Specific Cell HLA-DR and TBAg/PHA Ratio for Discriminating ATB From LTBI

We found both MTB-specific cell HLA-DR and TBAg/PHA ratio showed moderate performance in discrimination between ATB and LTBI. However, the overlap between MTB-specific cell HLA-DR and TBAg/PHA ratio was observed using Venn diagram analysis, suggesting the combination of these two indicators might further improve the diagnostic value (**Figures 2E, F**). To determine whether the combination of these two indicators could improve the ability to discriminate ATB from LTBI, we generated a diagnostic model using logistic regression. A diagnostic model was developed as the following: P = 1/[1 + e^-(0.094 × MTB-specific cell HLA-DR + 6.761 × TBAg/PHA ratio - 7.328)^] P, predictive value; e, natural logarithm. The diagnostic model yielded promising discriminatory potential with an AUC of 0.937 (95% CI, 0.892-0.982) (**Figures 2G, H**). When the threshold was set as 0.36, the model performed excellently with a sensitivity of 92.00% (95% CI, 81.16%-96.85%) and specificity of 81.82% (95% CI, 69.67%-89.81%) (**Table 2**).

### Value of Identified Biomarkers in Monitoring Anti-TB Treatment

**Figure 3:**
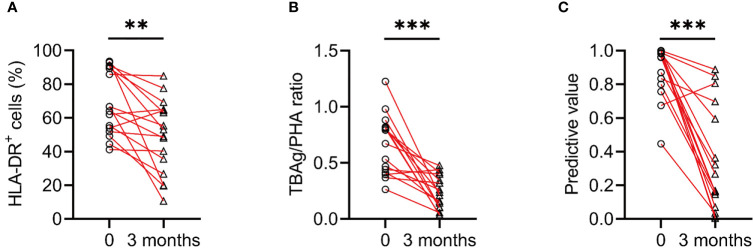
The change of various indicators after anti-TB treatment. **(A)** Line graphs showing the expression of HLA-DR on IFN-γ^+^TNF-α^+^ cells in ATB patients before and after 3 months of anti-TB treatment. **P < 0.01 (Wilcoxon test). **(B)** Line graphs showing the levels of TBAg/PHA ratio in ATB patients before and after 3 months of anti-TB treatment. ***P < 0.001 (Wilcoxon test). **(C)** Line graphs showing the predictive values of diagnostic model in ATB patients before and after 3 months of anti-TB treatment. ***P < 0.001 (Wilcoxon test). TBAg, tuberculosis antigens; PHA, phytohemagglutinin.

To evaluate whether these identified biomarkers have the potential to be used for TB treatment monitoring, we compared the levels of MTB-specific cell HLA-DR, TBAg/PHA ratio as well as predictive value of diagnostic model before and after anti-TB treatment. It was observed that the level of MTB-specific cell HLA-DR, TBAg/PHA ratio, and predictive value were all significantly decreased after standard anti-TB treatment (**Figure 3**).

### Independent Validation of the Diagnostic Model

**Figure 4:**
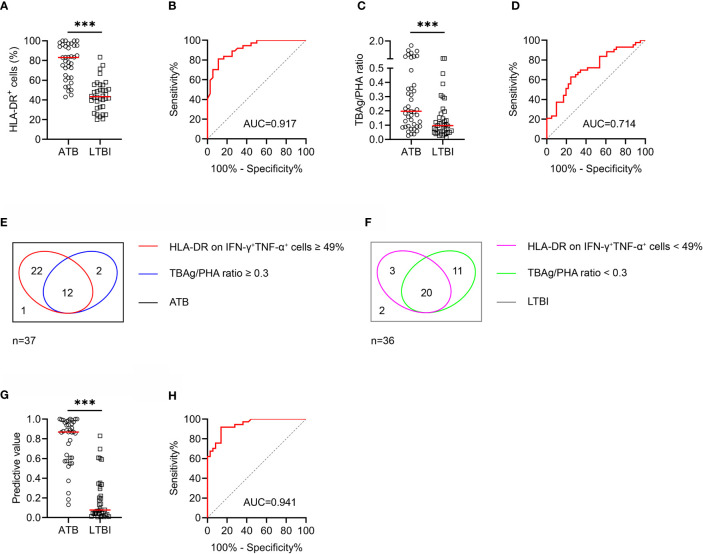
The performance of various indicators in distinguishing ATB patients from LTBI individuals in Caidian cohort. **(A)** Scatter dot plots showing the results of the expression of HLA-DR on IFN-γ^+^TNF-α^+^ cells in ATB patients and LTBI individuals. Horizontal lines indicate the medians. ***P < 0.001 (Mann-Whitney *U* test). **(B)** ROC curve analysis showing the performance of HLA-DR on IFN-γ^+^TNF-α^+^ cells in discriminating ATB patients from LTBI individuals. **(C)** Scatter dot plots showing the results of TBAg/PHA ratio in ATB patients and LTBI individuals. Horizontal lines indicate the medians. ***P < 0.001 (Mann-Whitney *U* test). **(D)** ROC curve analysis showing the performance of TBAg/PHA ratio in discriminating ATB patients from LTBI individuals. **(E)** Venn diagrams showing the overlap of HLA-DR on IFN-γ^+^TNF-α^+^ cells and TBAg/PHA ratio in ATB patients. **(F)** Venn diagrams showing the overlap of HLA-DR on IFN-γ^+^TNF-α^+^ cells and TBAg/PHA ratio in LTBI individuals. **(G)** Scatter plots showing the predictive value of diagnostic model in ATB patients and LTBI individuals. Horizontal lines indicate the medians. ***P < 0.001 (Mann-Whitney *U* test). **(H)** ROC curve analysis showing the performance of diagnostic model based on the combination of HLA-DR on MTB-specific cells and TBAg/PHA ratio in discriminating ATB patients from LTBI individuals. MTB, *Mycobacterium tuberculosis*; ATB, active tuberculosis; LTBI, latent tuberculosis infection; TBAg, tuberculosis antigens; PHA, phytohemagglutinin; AUC, area under the curve.

In order to validate the performance of the diagnostic model, another independent cohort was included for evaluation. Similar performance was obtained with HLA-DR on MTB-specific cells in Caidian cohort. MTB-specific cell HLA-DR distinguished ATB patients from LTBI individuals with an AUC of 0.917 (95% CI, 0.856-0.977) and demonstrated a sensitivity and specificity of 91.89% (95% CI, 78.70%-97.21%) and 63.89% (95% CI, 47.58%-77.53%), respectively (**Table 3** and **Figures 4A, B**). Meanwhile, TBAg/PHA ratio yielded a sensitivity of 37.21% (95% CI, 24.38%-52.14%) and specificity of 87.80% (95% CI, 74.46%-94.68%) in distinguishing ATB patients from LTBI individuals (**Table 3** and **Figures 4C, D**). Furthermore, the diagnostic model produced an AUC of 0.941 (95% CI, 0.893-0.989), with a sensitivity of 91.89% (95% CI, 78.70%-97.21%) and a specificity of 86.11% (95% CI, 71.34%-93.92%) when a threshold of 0.36 was used (**Table 3** and **Figures 4E–H**).

**Table 3:** The performance of various indicators for discriminating ATB from LTBI in Caidian cohort.

Variables	Cutoff value	AUC (95% CI)	Sensitivity (95% CI)	Specificity (95% CI)	PPV (95% CI)	NPV (95% CI)	PLR (95% CI)	NLR (95% CI)	Accuracy
HLA-DR on IFN-γ^+^TNF-α^+^ cells (%)	49	0.917 (0.856-0.977)	91.89% (78.70%-97.21%)	63.89% (47.58%-77.53%)	72.34% (58.24%-83.06%)	88.46% (71.03%-96.00%)	2.54 (1.63-3.97)	0.13 (0.04-0.39)	78.08%
TBAg/PHA ratio	0.3	0.714 (0.605-0.824)	37.21% (24.38%-52.14%)	87.80% (74.46%-94.68%)	76.19% (54.91%-89.37%)	57.14% (44.86%-68.60%)	3.05 (1.23-7.57)	0.72 (0.55-0.92)	61.90%
Diagnostic model	0.36	0.941 (0.893-0.989)	91.89% (78.70%-97.21%)	86.11% (71.34%-93.92%)	87.18% (73.30%-94.40%)	91.18% (77.04%-96.95%)	6.62 (2.92-15.01)	0.09 (0.03-0.28)	89.04%

ATB, active tuberculosis; LTBI, latent tuberculosis infection; TBAg, tuberculosis-specific antigen; PHA, phytohaemagglutinin; AUC, area under the curve; PPV, positive predictive value; NPV, negative predictive value; PLR, positive likelihood ratio; NLR, negative likelihood ratio; CI, confidence interval.

We also analyzed the pooled diagnostic performance of various indicators when combining two cohorts. It was observed that the sensitivity and specificity of MTB-specific cell HLA-DR for discriminating ATB from LTBI were 90.80% (95% CI, 82.89%-95.27%) and 62.64% (95% CI, 52.38%-71.88%), respectively (**Supplementary Table 1** and **Supplementary Figures 2A, B**). TBAg/PHA ratio distinguished ATB from LTBI with a sensitivity of 39.18% (95% CI, 30.05%-49.12%) and a specificity of 90.29% (95% CI, 83.05%-94.64%) (**Supplementary Table 1** and **Supplementary Figures 2C, D**). Moreover, the diagnostic model produced an AUC of 0.940 (95% CI, 0.907-0.972) in differentiating ATB from LTBI, with a sensitivity of 91.95% (95% CI, 84.31%-96.05%) and a specificity of 83.52% (95% CI, 74.57%-89.75%) (**Supplementary Table 1** and **Supplementary Figures 2E, F**).

## Discussion

Despite decades of research and significant investment, there still exists a huge gap in meeting clinical need for diagnosing TB. The conventional tools to diagnose ATB have major limitations including inadequate utility, high cost as well as long turnaround time (28). Meanwhile, an increasing number of studies denoted that host signature, especially omics, could serve as an alternative to pathogen detection (29). Identifying omics signatures for diagnosing TB have been facilitated by advances in technology to analyze obtained data using quantitative and high-throughput manner (30–33). However, even with numerous reports on novel candidates or multiple biomarker signatures, few of them have been independently validated for routine clinical use, and translated into applicable diagnostic tests. To achieve better management and control for TB, novel diagnostic strategies, especially based on present technology platform, are warranted.

Recently, MTB-specific cell signature such as activation and differentiation had been identified for the diagnosis of ATB (34–36). The available evidence indicated that HLA-DR on MTB-specific cells might be the most promising biomarker (37). Our data in this study also confirmed the discriminatory role of this indicator in distinguishing between ATB and LTBI. Notwithstanding, far less is known about the reliability and ability of combining MTB-specific cell HLA-DR and other indicators for the differential diagnosis between ATB and LTBI. To our knowledge, the present study established diagnostic model based on the combination of HLA-DR on MTB-specific cells and TBAg/PHA ratio for the first time. Our results demonstrated that the combination could further improve the diagnostic value. Moreover, we evaluated the value of these biomarkers in monitoring anti-TB treatment. The significant change with treatment denoted their potential value for monitoring therapy responses.

Two points should be pointed out in the present study. Firstly, we did find a high sensitivity and a moderate specificity of MTB-specific cell HLA-DR expression for differentiating ATB from LTBI. However, this seemed to be inconsistent with some previous studies, which showed that HLA-DR is a superior indicator with both excellent sensitivity and specificity (19). Nevertheless, we seemed to find an answer from the study conducted by Mpande and his/her colleagues (20). They reported that MTB-specific cells, from the LTBI population who were infected with MTB recently, also showed high activation, suggesting that the loss of specificity exhibited by HLA-DR in distinguishing ATB from LTBI is due to the presence of this population. Yet these cases did not show high values of TBAg/PHA ratio. Therefore, the combination of these two indicators could mainly contribute to improving the specificity, not the sensitivity. Second, we found that the sensitivity of TBAg/PHA ratio on distinguishing ATB from LTBI in this study was obviously lower than those reported in several previous studies (22), while similar with the utility obtained in a recent real-world data analysis from China (38) and another study from Japan (39). It was observed that the studies with good performance were often performed with patient exclusion such as immunosuppression. Therefore, the value of TBAg/PHA ratio in clinical application is mainly reflected in its acceptable specificity. In other words, TBAg/PHA ratio should be used more as a rule-in tool for its high specificity, rather than a rule-out test.

An optimal biomarker-based test would ideally be feasible with limited instrumentation and based on easily accessible samples such as peripheral blood. The methods involved in our diagnostic model fit the requirement. However, several limitations should be mentioned in this study. First, although two centers were included in our design, the number of subjects per center was too small and further validation with a large sample size is still needed in the future. Second, our model may not be helpful for T-SPOT-negative TB patients due to the lack of sufficient detectable MTB-specific cells in this population (40). More suitable tools should be developed targeting this population. Finally, since the established model was built based on two different techniques, the economic and clinical usefulness remain to be clarified in the further research.

In conclusion, the diagnostic model based on the combination of HLA-DR on MTB-specific cells and TBAg/PHA ratio would represent a new era of prompt TB diagnosis as an excellent auxiliary tool and enable earlier treatment, and thus reduce the spread of the disease, contributing toward paving the way for ending TB epidemic.

## Data Availability Statement

The original contributions presented in the study are included in the article/**Supplementary Material**. Further inquiries can be directed to the corresponding authors.

## Ethics Statement

The studies involving human participants were reviewed and approved by the Ethics Committee of Tongji Hospital, Tongji Medical College, Huazhong University of Science and Technology. The patients/participants provided their written informed consent to participate in this study.

## Author Contributions

YL: had the original idea of this study. YL: conceived and designed the study. YL, YX, BY, JH, and WW: performed the experiments. YL: performed data collection and analysis. YL: wrote the manuscript. YL, GT, QL, HS, WL, and LM: enrolled participants. YL, FW, and ZS: contributed to revisions of the manuscript. All authors contributed to the article and approved the submitted version.

## Funding

This work was funded by Graduate Innovation Fund of Huazhong University of Science and Technology (grant number 2021yjsCXCY088) and Special Foundation for National Science and Technology Basic Research Program of China (grant number 2019FY101206).

## Conflict of Interest

The authors declare that the research was conducted in the absence of any commercial or financial relationships that could be construed as a potential conflict of interest.

## Publisher’s Note

All claims expressed in this article are solely those of the authors and do not necessarily represent those of their affiliated organizations, or those of the publisher, the editors and the reviewers. Any product that may be evaluated in this article, or claim that may be made by its manufacturer, is not guaranteed or endorsed by the publisher.

## Supplementary Material

Supplementary Figure 1 The results of various indicators in different age range. **(A)** Scatter dot plots showing the results of the expression of HLA-DR on IFN-γ^+^TNF-α^+^ cells under young age (≤ 50 years old) and old age (> 50 years old) in ATB patients and LTBI individuals. Horizontal lines indicate the medians. **(B)** Scatter dot plots showing the results of TBAg/PHA ratio under young age (≤ 50 years old) and old age (> 50 years old) in ATB patients and LTBI individuals. Horizontal lines indicate the medians.

Supplementary Figure 2 The pooled performance of various indicators in distinguishing ATB patients from LTBI individuals. **(A)** Scatter dot plots showing the results of the expression of HLA-DR on IFN-γ^+^TNF-α^+^ cells in ATB patients and LTBI individuals. Horizontal lines indicate the medians. ****P* < 0.001 (Mann-Whitney *U* test). **(B)** ROC curve analysis showing the performance of HLA-DR on IFN-γ^+^TNF-α^+^ cells in discriminating ATB patients from LTBI individuals. **(C)** Scatter dot plots showing the results of TBAg/PHA ratio in ATB patients and LTBI individuals. Horizontal lines indicate the medians. ****P* < 0.001 (Mann-Whitney *U* test). **(D)** ROC curve analysis showing the performance of TBAg/PHA ratio in discriminating ATB patients from LTBI individuals. **(E)** Scatter plots showing the predictive value of diagnostic model in ATB patients and LTBI individuals. Horizontal lines indicate the medians. ****P* < 0.001 (Mann-Whitney *U* test). **(F)** ROC curve analysis showing the performance of diagnostic model based on the combination of HLA-DR on MTB-specific cells and TBAg/PHA ratio in discriminating ATB patients from LTBI individuals. MTB, *Mycobacterium tuberculosis*; ATB, active tuberculosis; LTBI, latent tuberculosis infection; TBAg, tuberculosis antigens; PHA, phytohemagglutinin; AUC, area under the curve.
