# Supplementary figures and images for: Combination of HLA-DR on Mycobacterium tuberculosis-Specific Cells and Tuberculosis Antigen/Phytohemagglutinin Ratio for Discriminating Active Tuberculosis From Latent Tuberculosis Infection
Source: Front Immunol. 2021 Nov 11;12:761209. doi: 10.3389/fimmu.2021.761209 (PMC8632229; doi:10.3389/fimmu.2021.761209)

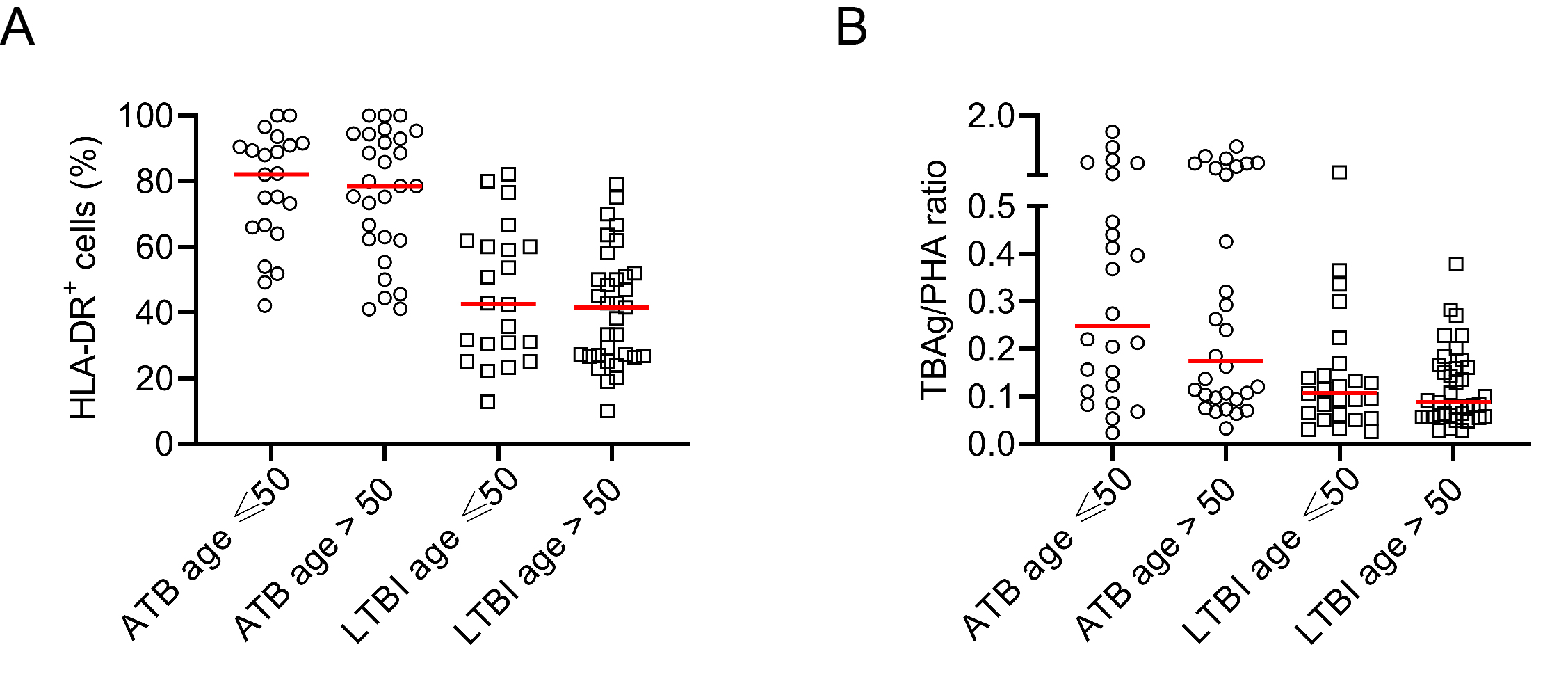

Supplement: Supplementary Figure 1 — The results of various indicators in different age range. (A) Scatter dot plots showing the results of the expression of HLA-DR on IFN-γ+TNF-α+ cells under young age (≤ 50 years old) and old age (> 50 years old) in ATB patients and LTBI individuals. Horizontal lines indicate the medians. (B) Scatter dot plots showing the results of TBAg/PHA ratio under young age (≤ 50 years old) and old age (> 50 years old) in ATB patients and LTBI individuals. Horizontal lines indicate the medians. [file Image_1.jpeg]

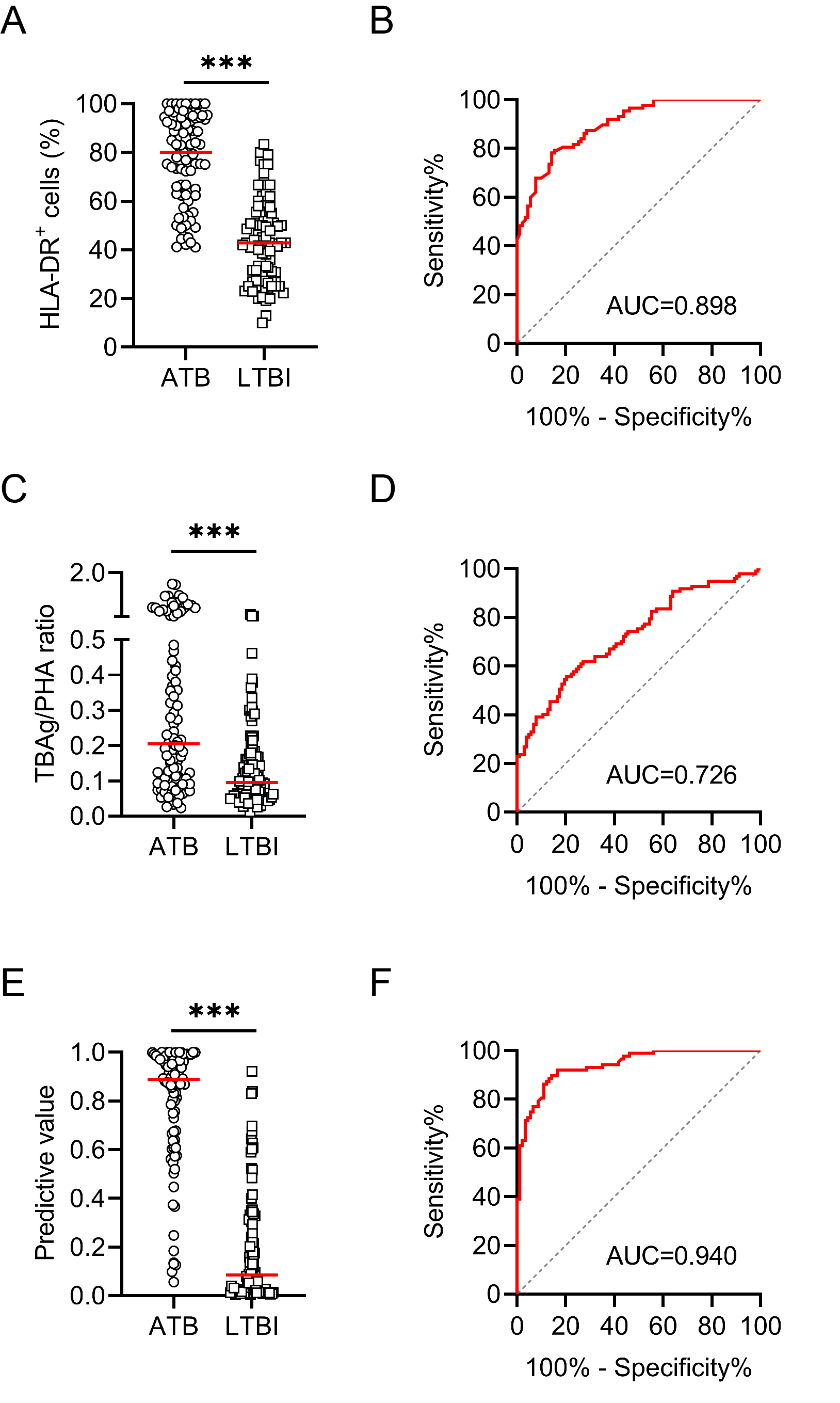

Supplement: Supplementary Figure 2 — The pooled performance of various indicators in distinguishing ATB patients from LTBI individuals. (A) Scatter dot plots showing the results of the expression of HLA-DR on IFN-γ+TNF-α+ cells in ATB patients and LTBI individuals. Horizontal lines indicate the medians. ***P < 0.001 (Mann-Whitney U test). (B) ROC curve analysis showing the performance of HLA-DR on IFN-γ+TNF-α+ cells in discriminating ATB patients from LTBI individuals. (C) Scatter dot plots showing the results of TBAg/PHA ratio in ATB patients and LTBI individuals. Horizontal lines indicate the medians. ***P < 0.001 (Mann-Whitney U test). (D) ROC curve analysis showing the performance of TBAg/PHA ratio in discriminating ATB patients from LTBI individuals. (E) Scatter plots showing the predictive value of diagnostic model in ATB patients and LTBI individuals. Horizontal lines indicate the medians. ***P < 0.001 (Mann-Whitney U test). (F) ROC curve analysis showing the performance of diagnostic model based on the combination of HLA-DR on MTB-specific cells and TBAg/PHA ratio in discriminating ATB patients from LTBI individuals. MTB, Mycobacterium tuberculosis; ATB, active tuberculosis; LTBI, latent tuberculosis infection; TBAg, tuberculosis antigens; PHA, phytohemagglutinin; AUC, area under the curve. [file Image_2.jpeg]
