# Supplementary material for: Combination of HLA-DR on Mycobacterium tuberculosis-Specific Cells and Tuberculosis Antigen/Phytohemagglutinin Ratio for Discriminating Active Tuberculosis From Latent Tuberculosis Infection
Source: Front Immunol. 2021 Nov 11;12:761209. doi: 10.3389/fimmu.2021.761209 (PMC8632229; doi:10.3389/fimmu.2021.761209)
Supplement: Supplementary file 3 [file Table_1.docx]

| **Supplementary Table 1. The pooled performance of various indicators for discriminating ATB from LTBI.** | | | | | | | | | |
| --- | --- | --- | --- | --- | --- | --- | --- | --- | --- |
| Variables | Cutoff value | AUC (95% CI) | Sensitivity (95% CI) | Specificity (95% CI) | PPV (95% CI) | NPV (95% CI) | PLR (95% CI) | NLR (95% CI) | Accuracy |
| HLA-DR on IFN-γ^+^TNF-α^+^ cells (%) | 49 | 0.898 (0.855-0.941) | 90.80% (82.89%-95.27%) | 62.64% (52.38%-71.88%) | 69.91% (60.91%-77.60%) | 87.69% (77.55%-93.63%) | 2.43 (1.85-3.2) | 0.15 (0.07-0.29) | 76.40% |
| TBAg/PHA ratio | 0.3 | 0.726 (0.657-0.796) | 39.18% (30.05%-49.12%) | 90.29% (83.05%-94.64%) | 79.17% (65.74%-88.27%) | 61.18% (53.25%-68.56%) | 4.04 (2.13-7.64) | 0.67 (0.57-0.8) | 65.50% |
| Diagnostic model | 0.36 | 0.940 (0.907-0.972) | 91.95% (84.31%-96.05%) | 83.52% (74.57%-89.75%) | 84.21% (75.57%-90.19%) | 91.57% (83.60%-95.86%) | 5.58 (3.5-8.9) | 0.1 (0.05-0.2) | 87.64% |
| ATB, active tuberculosis; LTBI, latent tuberculosis infection; TBAg, tuberculosis-specific antigen; PHA, phytohaemagglutinin; AUC, area under the curve; PPV, positive predictive value; NPV, negative predictive value; PLR, positive likelihood ratio; NLR, negative likelihood ratio; CI, confidence interval. | | | | | | | | | |
